# Difference between Toxicities of Iron Oxide Magnetic Nanoparticles with Various Surface-Functional Groups against Human Normal Fibroblasts and Fibrosarcoma Cells

**DOI:** 10.3390/ma6104689

**Published:** 2013-10-22

**Authors:** Won Jun Yang, Jong Ho Lee, Seong Cheol Hong, Jaewook Lee, Jaebeom Lee, Dong-Wook Han

**Affiliations:** World Class University Program, Department of Cogno-Mechatronics Engineering, Pusan National University, Busan 609-735, Korea; E-Mails: wjyang@sicns.com (W.J.Y.); pignunssob@naver.com (J.H.L.); snailcat@nate.com (S.C.H.); pizaalee@hotmail.com (J.L.)

**Keywords:** magnetic nanoparticles, surface functionalization, biocompatibility, normal cells, cancer cell

## Abstract

Recently, many nanomedical studies have been focused on magnetic nanoparticles (MNPs) because MNPs possess attractive properties for potential uses in imaging, drug delivery, and theranostics. MNPs must have optimized size as well as functionalized surface for such applications. However, careful cytotoxicity and genotoxicity assessments to ensure the biocompatibility and biosafety of MNPs are essential. In this study, Fe_3_O_4_ MNPs of different sizes (approximately 10 and 100–150 nm) were prepared with different functional groups, hydroxyl (–OH) and amine (–NH_2_) groups, by coating their surfaces with tetraethyl orthosilicate (TEOS), 3-aminopropyltrimethoxysilane (APTMS) or TEOS/APTMS. Differential cellular responses to those surface-functionalized MNPs were investigated in normal fibroblasts *vs.* fibrosarcoma cells. Following the characterization of MNP properties according to size, surface charge and functional groups, cellular responses to MNPs in normal fibroblasts and fibrosarcoma cells were determined by quantifying metabolic activity, membrane integrity, and DNA stability. While all MNPs induced just about 5% or less cytotoxicity and genotoxicity in fibrosarcoma cells at lower than 500 μg/mL, APTMS-coated MNPs resulted in greater than 10% toxicity against normal cells. Particularly, the genotoxicity of MNPs was dependent on their dose, size and surface charge, showing that positively charged (APTMS- or TEOS/APTMS-coated) MNPs induced appreciable DNA aberrations irrespective of cell type. Resultantly, smaller and positively charged (APTMS-coated) MNPs led to more severe toxicity in normal cells than their cancer counterparts. Although it was difficult to fully differentiate cellular responses to various MNPs between normal fibroblasts and their cancer counterparts, normal cells were shown to be more vulnerable to internalized MNPs than cancer cells. Our results suggest that functional groups and sizes of MNPs are critical determinants of degrees of cytotoxicity and genotoxicity, and potential mechanisms of toxicity.

## 1. Introduction

Nanomaterials that are defined as materials which have structured components with at least one dimension of 100 nm or less are increasingly being used for commercial purposes such as sensors, catalysts, cosmetics, electronic devices, drug carriers, imaging and therapeutic agents [1,2,3,4,5,6,7]. Toxicity and biosafety of medically used nanoscale materials have been argued frequently because materials in this size range may approach the length scale where some specific physicochemical interactions with their external species can occur. As a result, their properties differ substantially from those bulk materials of the same composition, allowing them to perform exceptional feats of conductivity, reactivity, and optical sensitivity [8]. Possible undesirable results of these capabilities are harmful interactions with biological systems and the environment, with the potential to generate toxicity [9,10,11]. In many pre-clinical studies about nanoparticles (NPs) toxicity, it would tend that the positive aspect of their designed therapeutic effects on cancer cell is emphasized usually while undesignated aspect to normal cell is subject to be underestimated, which may become a major bottleneck in the following experiments. For example, NP-based therapeutic/imaging systems may induce cytotoxicity or even genotoxicity, which may be different depending on cell types, or between normal cells and their cancer counterparts because the nano-system may alter physicochemical properties resulting in changes in their stability, solubility, surface charges and pH, and even pharmacokinetic disposition (in nano-drugs) in *in vitro* and *in vivo* environments [12]. Therefore, it is important to manage NP risk in both aspects, *i.e.*, the therapeutic effect and unknown side effect, by implementing a systematic process for identifying possible risks related to exposures to newly developed engineered nanomaterials.

In particular, superparamagnetic iron oxide NPs (SPIONs) are commercially used for *in vivo* applications such as a contrast agent for magnetic resonance imaging (MRI), for tumor therapy or cardiovascular disease diagnosis [13]. SPIONs are very promising NPs for these applications since they can be affected through external magnets. However, it is emerged that there is a plain need to identify any potential cellular damage associated with these NPs. Besides focusing on cytotoxicity, the most commonly used determinant of toxicity as a result of exposure to SPIONs, the importance of studying subtle cellular alterations should be mentioned in the form of DNA damage (*i.e.*, genotoxicity) and oxidative stress mediated by reactive oxygen species (ROS) [14,15,16]. Recently, it was found that the cell type can play quite a significant role in the definition of suitable pathways for detoxification of magnetic NPs (MNPs) [17,18,19]. In our previous study, we explained subtle changes in cytotoxicity and genotoxicity of SPIONs with different sizes and functional groups [20]. In the macroscopic view of the cell itself, SPIONs appear not to be cytotoxic and genotoxic to fibroblastic cells at higher concentrations (500 or 1000 μg/mL). However, it was obvious that the small modification of NPs induced subtle variations in their cellular internalization, or endocytosis. Furthermore, noticeable differences in the genotoxicity of different SPIONs, possibly due to variations in size and charge, were observed at low concentrations (≤200 μg/mL). In the present study, we aimed to determine differential cellular responses to surface-functionalized Fe_3_O_4_ MNPs of different sizes in human normal fibroblasts *vs.* fibrosarcoma cells and used the increasing concentration range (200–1000 μg/mL) of each MNP to examine at which concentration MNPs are toxic or not.

## 2. Results and Discussion

### 2.1. Physicochemical Characterizations of Fe_3_O_4_ MNPs

**Figure 1:**
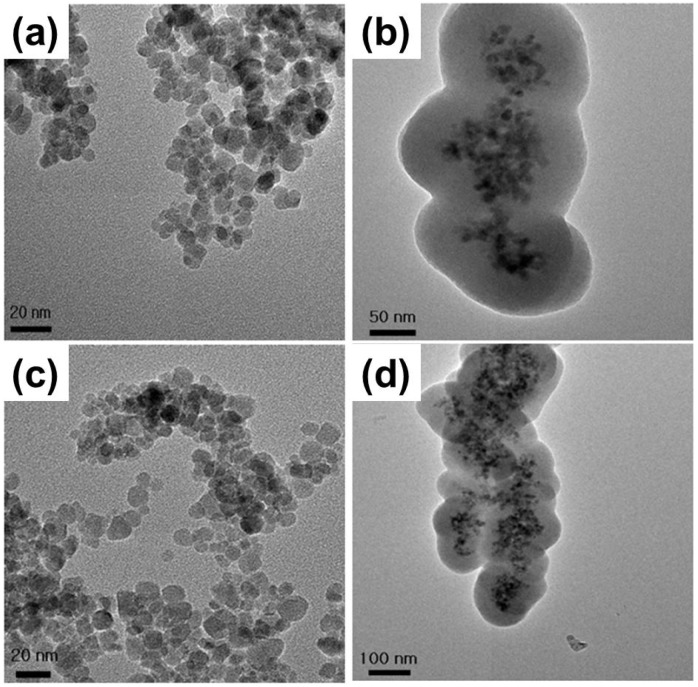
TEM images of (**a**) bare MNPs; (**b**) TEOS-coated MNPs; (**c**) APTMS-coated MNPs; and (**d**) TEOS/APTMS-coated MNPs.

MNP synthesis and surface modification were carefully carried out following the same methods as previously reported ones without any modification [20]. The morphology, size and shape of MNPs were observed by high-resolution transmission electron microscopy (HR-TEM) (Figure 1 and Table 1). In brief, bare MNPs were synthesized with an average diameter of about 10 nm. All subsequent surface and size modifications were initiated from these NPs. According to the type of surface coating agents, MNPs were modified with different functional groups; MNPs coated with tetraethylorthosilicate (TEOS) or TEOS/3-aminopropyltrimethoxysilane (APTMS) had the SiO_2_ shell, whereas MNPs with APTMS coating did not have it. Core MNPs were mostly similar in size because the nucleation and growth condition of NP synthesis were identical. After silication of NPs, a surface modification process was carried out using various functional groups, resulting in the formation of a single layer of the functional group on each MNP. The core material consisted of about five MNPs, and the thickness of the SiO_2_ shell was easily adjusted by altering TEOS molarity. The core/shell structure used in the toxicity evaluation had a core diameter of approximately 40 nm and an average shell thickness of 60 nm, yielding an average total diameter of 100–150 nm. Functional groups were hydroxyl or amine groups, which may yield strong negative and positive potentials, respectively, due to ionization in solution. When the surface of the MNPs was modified by these strong charged chemical groups, differently functionalized MNPs were dispersed well in phosphate-buffered saline (PBS) due to the high repulsion force among NPs. Zeta-potential analysis of surface potentials shows that all MNPs were charged at over ±30 mV, which is sufficient to be repulsive in PBS (pH 7.2) (Table 1). As both bare and silica-coated Fe_3_O_4_ MNPs show the negative surface charge, it seems that they have hydroxyl groups on their surface. It is considered that MNPs are not aggregated, but closely located to each other, since they possess magnetic properties and show single magnetic dipole behavior [21]. In addition, it was found that the hydrodynamic size of each MNP was slightly bigger than the size measured from the TEM image, implying that MNPs might agglomerate in the solution due to their magnetic property (Supplementary Figure S1).

**Table 1:** Physicochemical characteristics of magnetic nanoparticles (MNPs) tested: size and surface charge of each MNP.

Coating material	Average diameter (nm)	Surface charge (mV)	Functional group
none (bare)	10 ± 3	−20 ± 0.5	–O^−^
TEOS	100–150	−30 ± 1.8	–O^−^
APTMS	10 ± 4	25 ± 1.2	–NH_3_^+^
TEOS/APTMS	100–150	30 ± 2.0	–NH_3_^+^

### 2.2. Effects of MNPs on Metabolic Activity

**Figure 2:**
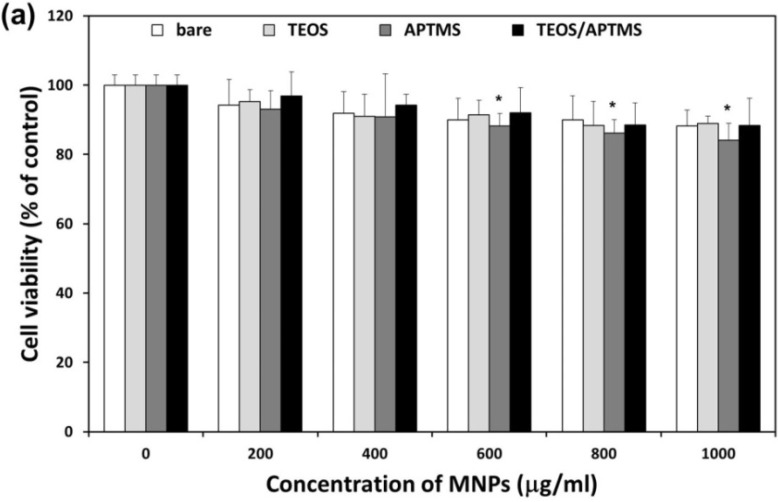

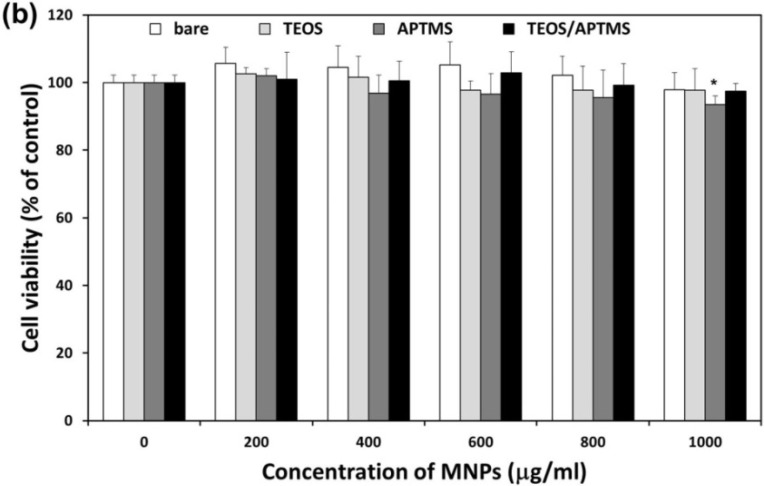
Effect of MNPs on metabolic activity. Cell viability of HDFs (**a**) and HT-1080 cells; (**b**) exposed for 24 h to increasing concentrations (0–1000 μg/mL) of MNPs coated with various functional groups was evaluated using the CCK-8 assay. * *p* < 0.05 *vs.* nontreated control.

After exposed to increasing concentrations (200–1000 μg/mL) of each MNP for 24 h, the cell viability of human dermal fibroblasts (HDFs) and human fibrosarcoma (HT-1080) cells was measured by using a cell counting kit-8 (CCK-8) assay (Figure 2). As all MNP solutions over 1000 μg/mL were saturated and easily precipitated, 1000 μg/mL was determined as the upper limit of concentration for MNP preparation. It is important to note that in almost all the studies, the toxicity is shown to increase significantly above a certain administration level [22]. Although high loadings (>100 μg/mL) of MNPs cause cytotoxicity, the concentrations needed for drug delivery and imaging applications are often below the toxic level for suitably coated MNPs [23]. Due to the physiological relevance of iron, MNPs were initially considered to be non-cytotoxic. MNPs can naturally be broken down resulting in the release of ferric iron, which can then participate in the normal iron metabolism [22]. It has, however, been recognized that the small size of MNPs might pose an additional hazard as the particles can reach high local concentrations within the cells and are generally more difficult to be efficiently cleared from the body [22,24]. Taking these things into consideration, the present study employed relatively high concentration ranges of MNPs to examine whether they are toxic or not at such doses although the ranges are not realistic compared to the commercial applications of MNPs.

As shown in Figure 2a, a slight reduction in the CCK-8 absorbance was observed in HDFs treated with increasing concentrations of each MNP in a dose-dependent manner. In particular, positively charged, APTMS-coated MNPs over 600 μg/mL more adversely affected the cell viability than other MNPs. Positively charged silicon NP–NH_2_ proved to be more cytotoxic in terms of reducing mitochondrial metabolic activity and effects on phagocytosis than neutral silicon NP–N_3_, while negatively charged silicon NP–COOH showed very little, or no, cytotoxicity [25]. On the contrary, all MNPs caused little significant reduction in the cell viability of HT-1080 cells within concentration ranges except for APTMS-coated MNPs at 1000 μg/mL and there was only some fluctuation (Figure 2b). A recent study reported that bare and oleic acid-coated Fe_3_O_4_ MNPs at 1000 μg/mL affected the viability of human hepatoma BEL-7402 cells via inducing cell arrest and apoptosis, and the MNPs-induced apoptosis was mediated through the mitochondrial-dependent pathway [26]. There are some explanations for the inconsistency, including difference in the cell type (fibrosarcoma HT-1080 cells *vs.* hepatocellular carcinoma BEL-7402 cells) and the determination method of cell viability (CCK-8 assay *vs.* WST-1 assay) as well as the coating material (TEOS and/or APTMS *vs.* oleic acid). Furthermore, it was revealed that poly(vinylalcohol)-coated SPIONs decreased antigen processing and CD4^+^ T cell stimulation capability of human monocyte-derived dendritic cells [27]. On the other hand, both dextran and polyethylene glycol coatings have been shown to reduce iron oxide NP cytotoxicity in aortic endothelial cells [28]. Our results suggest that the CCK-8 assay have any detection limit to find out differential cellular responses to surface-functionalized MNPs of different sizes in normal fibroblasts *vs.* fibrosarcoma cells. It is considered quite difficult for the CCK-8 assay to significantly differentiate effects of MNPs on the cell viability according to their surface functional groups because it depends on the activity of mitochondrial dehydrogenases [29]. Nevertheless, it was obviously revealed that HDFs were more vulnerable to internalized MNPs than HT-1080 cells. Previous studies show conflicting results on the toxicity of SPIONs; one study reported that there was no or low toxicity of SPIONs on human lung epithelial cell line (A549) at applied concentrations (40 and 80 μg/mL) [23], while in another study, they showed severe toxicity on human fibroblasts at the same concentration [30]. Additionally, SPIONs of various surface chemistries (bare, COOH, and NH_2_) showed different levels of cytotoxicity and gene expression patterns according to human cell types (*i.e.*, heart, brain, and kidney) [18].

### 2.3. Effects of MNPs on Cell Membrane Integrity

**Figure 3:**
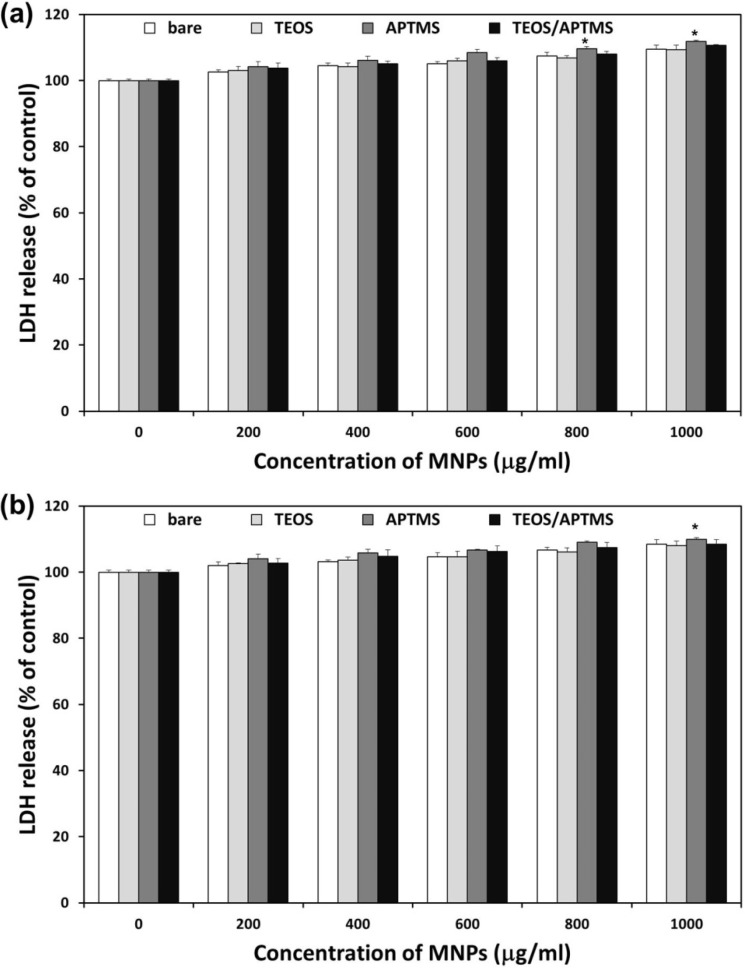
Effects of MNPs on cell membrane integrity. LDH release profiles in HDFs (**a**) and HT-1080 cells; (**b**) exposed to increasing concentrations (0–1000 μg/mL) of MNPs coated with various functional groups for 24 h were evaluated by the LDH assay. * *p* < 0.05 *vs.* nontreated control.

The amount of lactate dehydrogenase (LDH) released is proportional to the number of cells damaged or lysed and is a useful index for cytotoxicity based on the loss of membrane integrity [29]. All MNPs apparently induced LDH leakage from HDFs and HT-1080 cells 24 h after treatment, which implied adverse effects of MNPs on the cell membrane integrity (Figure 3). LDH levels in HDFs gradually increased as concentrations of MNPs increased (Figure 3a). Following exposure at the highest dose (1000 μg/mL) of positively charged, APTMS-coated and TEOS/APTMS-coated MNPs, LDH release from HDFs was about 112% and 111%, respectively. However, other negatively charged MNPs induced about 109% LDH release from cells. Resultantly, there was no significant difference among MNPs at the same concentration except for APTMS-coated MNPs at over 800 μg/mL. On the other hand, HT-1080 cells exhibited almost the same LDH release pattern as HDFs (Figure 3b). These results suggest that acute cytotoxicity might primarily result from the physical damage to the negatively charged cellular membrane particularly by positively charged MNPs. Nevertheless, there was little consistent evidence of cytotoxicity as the result of the CCK-8 assay. With the LDH assay itself, it seems very difficult to determine differential cellular responses to various MNPs in normal fibroblasts *vs.* their cancer counterparts since the influence of MNPs internalized into cells might be overlooked. Other study indicated that common *in vitro* cell endpoint assays, such as ROS production, lipid peroxidation and LDH leakage, should be compared with topography imaging by atomic force microscopy since they could not give detailed and complete information on cellular state [31]. Thus, it is essential to explore novel approaches and carry out more in-depth studies to elucidate cellular response mechanism to MNPs.

### 2.4. SEM and TEM Observations

**Figure 4:**
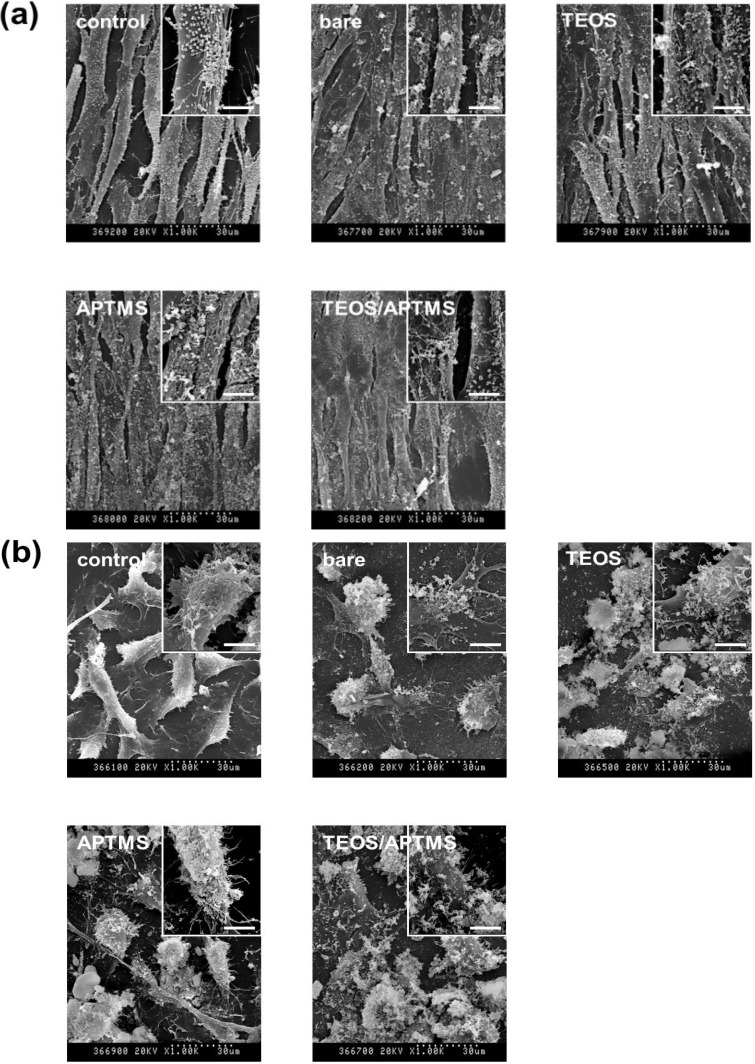

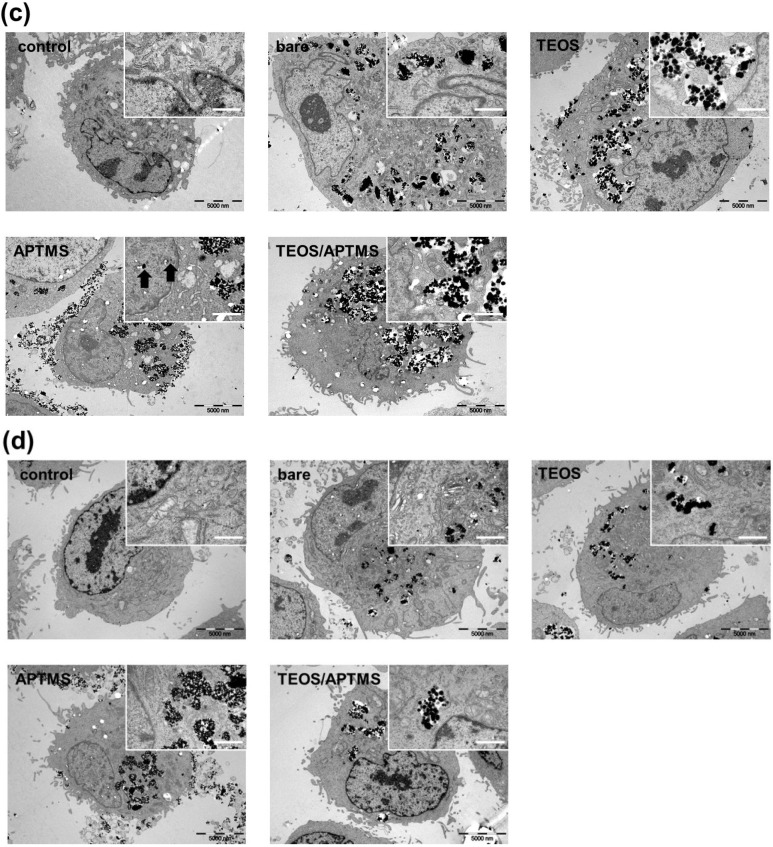
Electron microscopy images of (**a**,**c**) HDFs; and (**b**,**d**) HT-1080 cells. Morphological alterations and intracellular ultrastructures of both cells exposed to 500 μg/mL of MNPs coated with various functional groups for 24 h were observed by SEM (a,b; original magnification: ×1000) and TEM (c,d; original magnification: ×5000), respectively. Enlarged images of SEM (original magnification: ×5000, scale bar: 6 μm) and TEM (original magnification: ×30,000, scale bar: 1000 nm) are shown as the inset of each micrograph. The arrows in the inset of Figure 4c indicate APTMS-coated MNPs translocated into the nucleus of HDFs. All electron micrographs shown in this figure are representative of six independent experiments with similar results.

Morphological alterations of HDFs (Figure 4a) and HT-1080 cells (Figure 4b) were observed by scanning electron microscopy (SEM) after treatment with each MNP at a concentration of 500 μg/mL where cells were slightly damaged as indicated by CCK-8 and LDH assays. All MNPs were observed to be adhered to the surface of the cell membrane, which consists of negatively charged phospholipid bilayers, although it was expected that each MNP might show a different topograph according to its surface charge. Despite negative surface charges, bare and TEOS-coated MNPs were attached to the cell surface with local aggregation. APTMS-coated and TEOS/APTMS-coated MNPs, positively charged, appear to be more firmly attached to the membrane surface than negatively charged ones since the resting membrane potential is negative. Thus, it is suggested that the negatively charged membrane preferentially attracts positively charged, rather than negatively charged particles. Both cells treated with MNPs were shown to have almost similar morphologies to their nontreated controls. This result well agreed with that of the LDH assay indicating that attached particles showed a few adverse effects on the cell viability, which implies that MNPs rarely affected cell membrane integrity.

TEM observation provided essential information regarding the intracellular ultrastructure and detailed cellular uptake of MNPs into HDFs (Figure 4c) and HT-1080 cells (Figure 4d). The cellular incorporation of MNPs is highly related to their coating material’s charge and size. TEM images revealed that positively charged (APTMS- and TEOS/APTMS-coated) MNPs seemed to be more concentrated inside cells than negatively charged (bare and TEOS-coated) MNPs. It is considered that cells were more prone to damages by oxidative stress resulted from intracellular concentrated MNPs [32]. Particularly, large amounts of APTMS-coated MNPs widely adhered to the immediate vicinity of the cell membrane and several particles translocated even into the cell nucleus (the inset of Figure 4c). This result was favorably supported by a recent study reporting that positively charged SPION-NH_2_ and SPION-SH demonstrated a significant amount of uptake compared to negatively charged SPION-COOH and SPION-OH [33]. On the other hand, more intracellular vesicles containing positively charged MNPs were observed inside HDFs after exposure to those MNPs compared with HT-1080 cells. This observation may account partly for differential cellular responses to surface-functionalized MNPs of different sizes in normal fibroblasts *vs.* fibrosarcoma cells. Mechanisms for the cellular uptake of NPs are very complex and considered as endocytotic pathways such as phagocytosis, pinocytosis, nonspecific endocytosis, and receptor-mediated endocytosis [34,35]. Generally, the endocytosis of NPs depends on the interaction between NPs and the cell membrane. After the membrane is wrapped by NPs, some NPs are invaginated in the vesicle, which looks like agglomerates [36]. Although the further detailed study for the cellular uptake mechanism was not examined here, previous seminal studies have shown that larger NPs (100–200 nm) would be incorporated into cells more readily by endocytosis than smaller ones (~10 nm), which are generally taken up by cells via pinocytosis [37,38]. Smaller APTMS-coated MNPs are considered to easily permeate the cell membrane by pinocytosis. In contrast, larger TEOS- or TEOS/APTMS-coated MNPs seem to penetrate into cells via nonspecific endocytosis since these MNPs are about 100 nm in diameter and have no specific ligands for receptors on the cell membrane. Different cellular responses to various MNPs between normal fibroblasts and their cancer counterparts may therefore be elucidated based on the TEM observation although it cannot provide a quantitative analysis. On the contrary, CCK-8 and LDH assays can make it possible that cytotoxicity is quantitatively determined regardless of the tendency of MNPs to be internalized into cells while not properly reflect the cellular uptake of MNPs as observed by the TEM. Nanomaterials induce cell specific responses resulting in variable toxicity and subsequent cell fate based on the type of exposed cell, and the composition and size of nanomaterials as well as the target cell type are critical determinants of intracellular responses, degree of cytotoxicity and potential mechanisms of toxicity [39].

### 2.5. Effects of MNPs on DNA Stability

**Figure 5:**
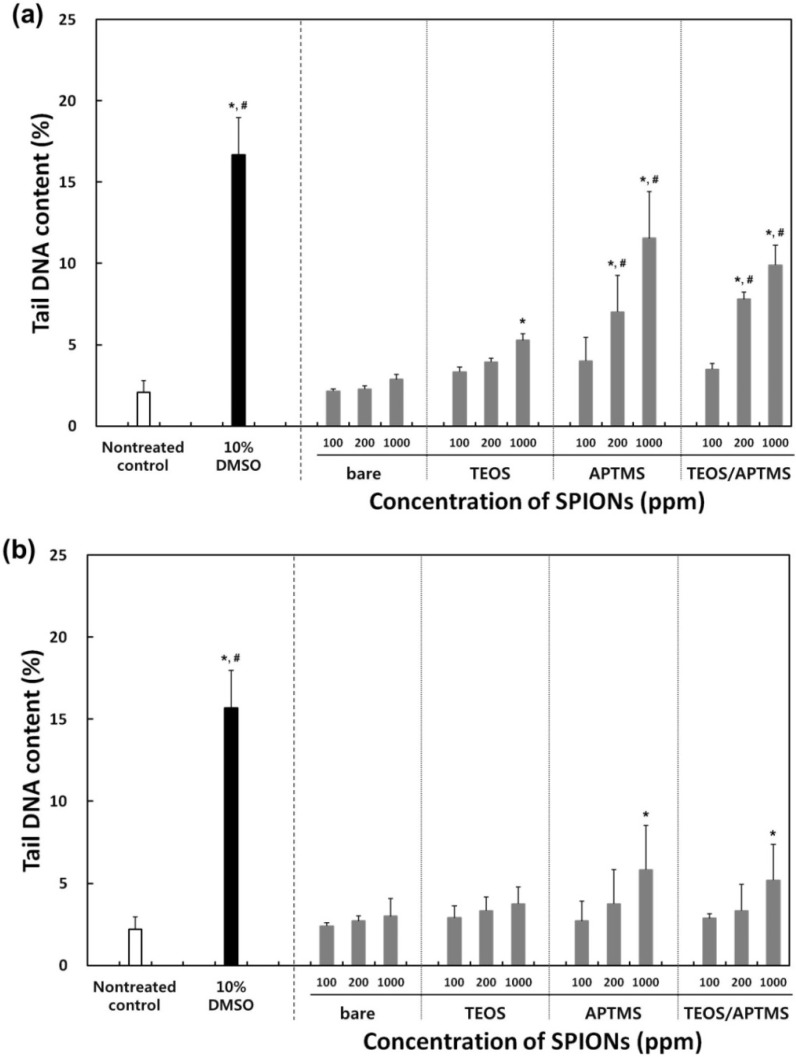
Effects of MNPs on DNA stability. Tail content of DNA in (**a**) HDFs; and (**b**) HT-1080 cells exposed to increasing concentrations (0–1000 μg/mL) of MNPs coated with various functional groups for 24 h was determined by the comet assay. * *p* < 0.05 *vs.* nontreated control, ^#^ *p* < 0.05 *vs.* MNP-treated cells with 100 μg/mL.

In order to determine differential effects of MNPs on the DNA stability in normal fibroblasts *vs.* fibrosarcoma cells, both cells were treated with MNPs at the concentrations of 100, 200, and 1000 μg/mL, or with 10% dimethyl sulfoxide (DMSO) as a positive control. After MNP treatments, both cells showed dose-dependent increases in the tail momentum compared to nontreated controls (Figure 5). The tendency towards genotoxicity of MNPs in normal cells was apparently similar to that of cancer cells, but there was appreciable difference between them. Bare and TEOS-coated MNPs resulted in neither extensive nor dose-dependent damages to the DNA stability in both cells. Tail DNA contents in cells treated with bare and TEOS-modified MNPs were lower than 5% even at 1000 μg/mL, which slightly exceeded the level (2%) that was seen with the nontreated (negative) control, but was still lower than that (16%) seen with the positive control (DMSO). In particular, MNPs modified with APTMS and TEOS/APTMS resulted in significant dose-dependent genotoxicity against normal cells (Figure 5a). It is just hypothesized that positively charged particles, APTMS-coated MNPs, having small sizes (~10 nm), are more prone to enter the nucleus through nuclear pores (about 10 nm in diameter) and interact directly with the chromosomal DNA, which is negatively charged due to the phosphate backbone. Although TEOS/APTMS-coated MNPs cannot be translocated into the nucleus, they induce indirect DNA damages via oxidative stress mediated by ROS resulted from the intracellular MNPs. In the case of fibrosarcoma cells, these positively charged MNPs induced appreciable DNA aberrations only at 1000 μg/mL (Figure 5b). These results well agreed with trends seen in CCK-8 and LDH assays. From these results, it is suggested that differential cellular responses to various MNPs between normal fibroblasts and their cancer counterparts can be determined based on the comet assay, which might quantitatively support qualitative results from the TEM.

The genotoxic mechanism may be revealed from further consecutive studies. It is generally noted from the experimental data that the higher the MNP concentration, the lower the cell viability and the worse the genotoxicity. Based on results of the genotoxicity assay, it is predicted that the toxicity of MNPs might result in DNA damage via oxidation of MNPs. It has been reported that SPIONs show little toxicity at 80 μg/mL despite their potency to cause DNA lesions in A549 human lung epithelial cells [23]. Our results also indicate that MNPs rarely show genotoxicity below at 100 μg/mL, but more careful investigation is required when MNPs are treated at concentrations above 100 μg/mL. For clinical labeling of cells in MRI, commercialized SPIONs (Resovist^®^, Schering, Germany) are currently used at concentrations of 5–25 μg/mL depending on the patient’s weight [40,41]; this concentration range is biologically safe based on our data from Figure 2, Figure 3 and Figure 4. Although only dextran-coated SPIONs are approved for human *in vivo* use by the Food and Drug Administration [42], several SPIONs with various physicochemical properties are in clinical trials and will be utilized for various biomedical applications in the near future [43]. Moreover, a fundamentally different approach to surface fabrication is essential to increase the biocompatibility of MNP since aggregation of MNP in biological milieu in the presence of strong static magnetic field from MRI is inevitable [44]. Eventually, to ensure the biosafety and biocompatibility of the MNPs, systematic and standardized cytotoxicity and genotoxicity assessments are strongly required.

## 3. Experimental Section

### 3.1. Synthesis and Characterizations of Surface-Functionalized MNPs

#### 3.1.1. Bare MNPs

MNPs were synthesized using the Massart’s method, based on the coprecipitation of ferrous and ferric ion solutions (1:2 molar ratios) [18]. In brief, 5 mL NH_4_OH (28–30 wt %, Sigma-Aldrich, St. Louis, MO, USA) was added to 10 mmol FeCl_3_ (97%, Sigma-Aldrich) and 5 mmol FeCl_2_ (99%, Wako Pure Chemical, Osaka, Japan) in 40 mL distilled water (DW) under rapid mechanical stirring at room temperature. Stirring was allowed to continue for 30 min, during which a black precipitate was formed. The precipitate was separated by magnetic decantation, washed three times with 20 mL ethanol, and then air-dried at room temperature. Their diameter was approximately 10 nm with a narrow size distribution. Immediately, the surface modification of the core MNPs was carried out to accomplish different sizes and surface functionalities as shown in Table 1.

#### 3.1.2. TEOS-Coated MNPs

Core/shell MNPs were synthesized according to the method of Stöber [17,19]. In brief, 25 mg MNPs were redispersed into a mixture of 5 mL ammonium hydroxide, 59.25 mL ethanol and 25 mL DW by sonication. An ethanolic solution (10 mL) of 0.5 mL TEOS (99%, Sigma-Aldrich) was added with mechanical stirring. The hydrolysis and condensation of TEOS onto MNPs were completed in 4 h. These particles were refined by magnetic precipitation, washed three times with 20 mL ethanol, and air-dried at room temperature. Core/shell spheres had an average diameter of 100 nm (±10 nm).

#### 3.1.3. APTMS-Coated MNPs

Amino-functionalized MNPs were synthesized according to a previously reported protocol [45]. Briefly, 25 mg MNPs were redispersed in 100 mL DW and ethanol mixture (3:7) by sonication. Then, 70 μL APTMS (>97%, Sigma-Aldrich) was added with vigorous stirring, and the solution was kept stirring at room temperature overnight. The black precipitate formed was purified by magnetic precipitation, washed three times with 20 mL ethanol, and air-dried at room temperature.

#### 3.1.4. TEOS/APTMS-Coated MNPs

The same method was used as described in the synthesis of APTMS-coated MNPs, except that TEOS-coated MNPs were used instead of bare MNPs as the initial core material, to prepare MNPs of a different size with different functional groups. The detailed characteristics and differences between the different MNPs are schematically described in Table 1.

#### 3.1.5. Physicochemical Characterizations

The average particle size and shape of each MNP were characterized by HR-TEM (JEOL, JEM-3010, Boston, MA, USA). Surface potentials of synthesized MNPs were measured using a Zetasizer (Nano ZS, Malvern Instruments, Worcestershire, UK).

### 3.2. Cell Cultures and Conditions

HDFs from neonatal dermis were kindly provided by Dr. Dong Kyun Rah (Department of Plastic and Reconstructive Surgery, Yonsei University College of Medicine, Seoul, Korea). Human fibrosarcoma (HT-1080) cells were purchased from American Type Culture Collection (ATCC CCL-121™, Rockville, MD, USA). Both cells were routinely maintained in Dulbecco’s modified Eagle’s medium (Sigma-Aldrich) supplemented with 10% fetal bovine serum (Sigma-Aldrich) and a 1% antibiotic antimycotic solution (including 10,000 units penicillin, 10 mg streptomycin and 25 μg amphotericin B per mL, Sigma-Aldrich) at 37 °C in a humidified atmosphere of 5% CO_2_ in air. Studies were performed with HDFs and HT-1080 cells within 10 and 25 passages, respectively.

### 3.3. CCK-8 Assay for Cytotoxicity Determination

The number of viable cells was indirectly quantified by using the CCK-8 assay (Dojindo, Kumamoto, Japan), which contains highly water-soluble tetrazolium salt [WST-8, 2-(2-methoxy-4-nitrophenyl)-3-(4-nitrophenyl)-5-(2,4-disulfophenyl)-2*H*-tetrazolium, monosodium salt], reduced to a yellow-color formazan dye by activities of dehydrogenases in cells. The cell viability is well-known to be directly proportional to metabolic reaction products obtained in the CCK-8 assay. Briefly, this assay was conducted as follows. HDFs and HT-1080 cells were treated with increasing concentrations (200–1000 μg/mL) of each MNP, and then incubated with CCK-8 for the last 4 h of the culture period (24 h) at 37 °C in the dark. In order to avoid a direct reaction between MNPs and WST-8, excess particles were completely removed thru washing with Dulbecco’s PBS (DPBS, Sigma-Aldrich), and the medium was exchanged before adding WST-8. Parallel sets of wells containing freshly cultured non-treated cells were regarded as negative controls. The absorbance was determined at 450 nm using an ELISA reader (SpectraMax^®^ 340, Molecular Device Co., Sunnyvale, CA, USA). The relative cell viability was determined as the percentage ratio of the optical density in the medium (containing MNPs at each concentration) to that of fresh control medium.

### 3.4. LDH Assay for Cytotoxicity Determination

Cell membrane integrity was monitored by using the LDH assay (Takara Bio Inc, Shiga, Japan), which can determine the release of LDH into the medium [46], according to manufacturer instructions. In this assay, LDH released from damaged cells oxidizes lactate to pyruvate, which promotes conversion of tetrazolium salt INT to a water-soluble red formazan product. Briefly, after 24 h exposure to increasing concentrations (200–1000 μg/mL) of each MNP, the supernatant from each well was transferred to a new 96-well plate. Reconstituted substrate mix was added to each well and plates were kept for 30 min in the dark at room temperature. Stop solution was added to each well. Parallel sets of wells containing freshly cultured cells were regarded as negative controls. Released LDH catalyzed the oxidation of lactate to pyruvate with simultaneous reduction of NAD^+^ to NADH. The rate of NAD^+^ reduction was measured as an increase in the absorbance at 340 nm and was directly proportional to the LDH activity in the cell medium. The intensity of red color formed in the assay and measured at a wavelength of 490 nm with an ELISA reader (SpectraMax^®^ 340, Molecular Device Co.), was proportional to the LDH activity and to the number of damaged cells.

### 3.5. Electron Microscopy Observations

Morphological alterations of HDFs and HT-1080 cells treated with 500 μg/mL of each MNP were observed by SEM. In brief, MNP-treated cells were washed with 0.1 M cacodylate buffer (pH 7.4) to remove unattached cells. Cells were fixed with 2.5% glutaraldehyde solution overnight at 4 °C, dehydrated with a series of increasing concentration of an ethanol solution, and then vacuum-dried. Fixed cell cultures were coated with an ultra-thin layer of gold/platinum by an ion sputter (E1010, Hitachi, Tokyo, Japan), and then observed by SEM (Hitachi S-800) at an accelerating voltage of 20 kV.

TEM was also performed to obtain information regarding the intracellular ultrastructure and distribution of MNPs taken up by cells. After being treated with each MNP for 24 h, cells were immediately fixed with 2% glutaraldehyde, rinsed twice with DPBS, and then post-fixed in 1% sodium cacodylate-buffered osmium tetroxide (OsO_4_). Fixed cell cultures were subsequently dehydrated through a graded series of ethanol solutions and finally embedded *in situ* by covering with a layer of Spurr epoxy resin (Polysciences Inc., Warrington, PA, USA), which was allowed to polymerize. Prepared blocks were sectioned using a diamond knife mounted in an ultracut-Reichert microtome (Leica, Heidelberg, Germany). Ultrathin sections (70–80 nm) were contrasted with uranyl acetate and lead citrate, and observed using an electron microscope (CM-120, Philips, Eindhoven, The Netherlands) at 80 keV.

### 3.6. Comet Assay for Genotoxicity Evaluation

The comet assay was performed essentially following the same method as reported elsewhere with some modification [47,48]. All reagents used were analytical grade (Sigma-Aldrich) unless otherwise stated and the entire process was performed in low light conditions to prevent induced DNA damage. Clear glass slides were pre-coated with 1% agarose (normal melting point). All slides were shielded from UV light during preparation and analysis. HDFs and HT-1080 cells were exposed to 100, 200 and 1000 μg/mL of each MNP for 24 h and then mildly trypsinized. In order to prevent potential interference of MNPs with the subsequent process, cells were thoroughly washed with DPBS prior to trypsinization. Parallel sets of wells containing freshly cultured cells and 10% DMSO-treated cells were regarded as negative and positive controls, respectively. An aliquot of the counted cell suspension, sufficient to provide approximately 30,000 cells per gel, was centrifuged (180× *g*, 5 min at 4 °C) and the supernatant were carefully removed. The cell pellet was then suspended in warm (37 °C) 0.6% agarose (low melting point) and two aliquots placed onto a glass slide. Each aliquot was then immediately overlaid with a cover slip. Rapid solidification of the agarose was achieved by placing slides on a metal tray on ice for 5 min. Cover slips were then carefully removed, and slides were placed in lysis buffer (100 mM EDTA, 2.5 M NaCl, 10 mM Tris–HCl, and 1% Triton X-100, adjusted to pH 10 with NaOH) and left overnight. Following cell lysis, slides were washed twice by submersion in ice-cold deionized water, then transferred to an electrophoresis tank, containing cold electrophoresis buffer (300 mM NaOH and 1 mM EDTA, pH 13), and incubated for 20 min to allow unwinding of the DNA. Electrophoresis was carried out for 20 min at 30 V and 300 mA. Slides were removed from the tank and flooded with neutralization buffer (0.4 M Tris–HCl, pH 7.5), then rinsed twice with deionized water. After neutralization, slides were kept in a humid atmosphere in a dark box at 4 °C until further analysis. For image analysis, DNA was stained with 2 μg/mL of ethidium bromide solution per slide for 10 min. Comets were examined under a fluorescence microscope (IX81-F72, Olympus Optical Co, Osaka, Japan). Only comets with a defined head were scored. Comet parameters considered in this study were the tail length and the proportion of DNA in the comet tail (tail DNA or tail intensity), which was calculated as the product of the fraction of DNA in the comet tail and the tail length [49].

### 3.7. Statistical Analysis

All variables were tested in three independent cultures for cytotoxicity and genotoxicity assays, which was repeated twice (*n* = 6). Results of cytotoxicity and genotoxicity are expressed as the mean ± standard deviation (SD) compared with nontreated controls. A one-way analysis of variance (ANOVA, SAS Institute Inc., Cary, NC, USA), which was followed by a Tukey honestly significant difference test for multiple comparisons, was used to detect dose-dependent effects of MNPs on HDFs and HT-1080 cells. A *P*-value < 0.05 was considered statistically significant.

## 4. Conclusions

Although it was difficult to fully differentiate cellular responses to various MNPs between normal fibroblasts and their cancer counterparts, normal cells were shown to be more vulnerable to internalized MNPs than cancer cells. It was obvious that small modifications in NPs might induce slight but essentially differential changes of cytotoxicity and genotoxicity between normal fibroblasts and their cancer counterparts; this information would be significantly valuable in possible applications of MNPs for drug delivery, cell imaging, cancer-targeting, or theragnosis requiring further advanced precise control.

## References

[1] Frey N.A., Peng S., Cheng K., Sun S. (2009). Magnetic nanoparticles: Synthesis, functionalization, and applications in bioimaging and magnetic energy storage. Chem. Soc. Rev..

[2] Gao J., Gu H., Xu B. (2009). Multifunctional magnetic nanoparticles: Design, synthesis, and biomedical applications. Acc. Chem. Res..

[3] Frimpong R.A., Hilt J.Z. (2010). Magnetic nanoparticles in biomedicine: Synthesis, functionalization and applications. Nanomedicine.

[4] Lee J., Kim H.Y., Zhou H., Hwang S., Koh K., Han D.-W., Lee J. (2011). Green synthesis of phytochemical-stabilized Au nanoparticles under ambient conditions and their biocompatibility and antioxidative activity. J. Mater. Chem..

[5] Granitzer P., Rumpf K. (2011). Magnetic nanoparticles embedded in a silicon matrix. Materials.

[6] Yu M.K., Park J., Jon S. (2012). Targeting strategies for multifunctional nanoparticles in cancer imaging and therapy. Theranostics.

[7] Wahajuddin, Arora S. (2012). Superparamagnetic iron oxide nanoparticles: Magnetic nanoplatforms as drug carriers. Int. J. Nanomedicine.

[8] Auffan M., Rose J., Bottero J.Y., Lowry G.V., Jolivet J.P., Wiesner M.R. (2009). Towards a definition of inorganic nanoparticles from an environmental, health and safety perspective. Nat. Nanotechnol..

[9] Nel A., Xia T., Mädler L., Li N. (2006). Toxic potential of materials at the nanolevel. Science.

[10] Murphy C.J., Gole A.M., Stone J.W., Sisco P.N., Alkilany A.M., Goldsmith E.C., Baxter S.C. (2008). Gold nanoparticles in biology: Beyond toxicity to cellular imaging. Acc. Chem. Res..

[11] Boisselier E., Astruc D. (2009). Gold nanoparticles in nanomedicine: Preparations, imaging, diagnostics, therapies and toxicity. Chem. Soc. Rev..

[12] Vega-Villa K.R., Takemoto J.K., Yáñez J.A., Remsberg C.M., Forrest M.L., Davies N.M. (2008). Clinical toxicities of nanocarrier systems. Adv. Drug Deliv. Rev..

[13] Neuberger T., Schopf B., Hofmann H., Hofmann M., von Rechenberg B. (2005). Superparamagnetic nanoparticles for biomedical applications: possibilities and limitations of a new drug delivery system. J. Magn. Magn. Mater..

[14] Mbeh D.A., França R., Merhi Y., Zhang X.F., Veres T., Sacher E., Yahia L. (2012). *In vitro* biocompatibility assessment of functionalized magnetite nanoparticles: Biological and cytotoxicological effects. J. Biomed. Mater. Res. A.

[15] Novotna B., Jendelova P., Kapcalova M., Rossner P., Turnovcova K., Bagryantseva Y., Babic M., Horak D., Sykova E. (2012). Oxidative damage to biological macromolecules in human bone marrow mesenchymal stromal cells labeled with various types of iron oxide nanoparticles. Toxicol. Lett..

[16] Singh N., Jenkins G.J., Nelson B.C., Marquis B.J., Maffeis T.G., Brown A.P., Williams P.M., Wright C.J., Doak S.H. (2012). The role of iron redox state in the genotoxicity of ultrafine superparamagnetic iron oxide nanoparticles. Biomaterials.

[17] Kumari A., Kumar V., Yadav S.K. (2011). Therapeutic nanoparticles and associated toxicity. Curr. Nanosci..

[18] Mahmoudi M., Laurent S., Shokrgozar M.A., Hosseinkhani M. (2011). Toxicity evaluations of superparamagnetic iron oxide nanoparticles: Cell “vision” *versus* physicochemical properties of nanoparticles. ACS Nano.

[19] Sharifi S., Behzadi S., Laurent S., Forrest M.L., Stroeve P., Mahmoudi M. (2012). Toxicity of nanomaterials. Chem. Soc. Rev..

[20] Hong S.C., Lee J.H., Lee J., Kim H.Y., Park J.Y., Cho J., Lee J., Han D.-W. (2011). Subtle cytotoxicity and genotoxicity differences in superparamagnetic iron oxide nanoparticles coated with various functional groups. Int. J. Nanomedicine.

[21] Zhang F., Wang C.C. (2008). Fabrication of one-dimensional iron oxide/silica nanostructures with high magnetic sensitivity by dipole-directed self-assembly. J. Phys. Chem. C.

[22] Bucak S., Yavuztürk B., Sezer A.D., Ali D.S. (2012). Magnetic nanoparticles: synthesis, surface modifications and application in drug delivery. Recent Advances in Novel Drug Carrier Systems.

[23] Karlsson H.L., Cronholm P., Gustafsson J., Möller L. (2008). Copper oxide nanoparticles are highly toxic: a comparison between metal oxide nanoparticles and carbon nanotubes. Chem. Res. Toxicol..

[24] Rivera G.P., Huhn D., del Mercato L.L., Sasse D., Parak W.J. (2010). Nanopharmacy: Inorganic nanoscale devices as vectors and active compounds. Pharmacol. Res..

[25] Bhattacharjee S., de Haan L.H., Evers N.M., Jiang X., Marcelis A.T., Zuilhof H., Rietjens I.M., Alink G.M. (2011). Role of surface charge and oxidative stress in cytotoxicity of organic monolayer-coated silicon nanoparticles towards macrophage NR8383 cells. Part Fibre Toxicol..

[26] Kai W., Xiaojun X., Ximing P., Zhenqing H., Qiqing Z. (2011). Cytotoxic effects and the mechanism of three types of magnetic nanoparticles on human hepatoma BEL-7402 cells. Nanoscale Res. Lett..

[27] Blank F., Gerber P., Rothen-Rutishauser B., Sakulkhu U., Salaklang J., de Peyer K., Gehr P., Nicod L.P., Hofmann H., Geiser T., Petri-Fink A., von Garnier C. (2011). Biomedical nanoparticles modulate specific CD4^+^ T cell stimulation by inhibition of antigen processing in dendritic cells. Nanotoxicology.

[28] Yu M., Huang S., Yu K.J., Clyne A.M. (2012). Dextran and polymer polyethylene glycol (PEG) coating reduce both 5 and 30 nm iron oxide nanoparticle cytotoxicity in 2D and 3D cell culture. Int. J. Mol. Sci..

[29] Lewinski N., Colvin V., Drezek R. (2008). Cytotoxicity of nanoparticles. Small.

[30] Gupta A.K., Wells S. (2004). Surface-modified superparamagnetic nanoparticles for drug delivery: Preparation, characterization, and cytotoxicity studies. IEEE Trans. Nanobiosci..

[31] Hoskins C., Cuschieri A., Wang L. (2012). The cytotoxicity of polycationic iron oxide nanoparticles: Common endpoint assays and alternative approaches for improved understanding of cellular response mechanism. J. Nanobiotechnol..

[32] Singh N., Jenkins G.J., Asadi R., Doak S.H. (2010). Potential toxicity of superparamagnetic iron oxide nanoparticles (SPION). Nano Rev..

[33] Bhattacharya D., Sahu S.K., Banerjee I., Das M., Mishra D., Maiti T.K., Pramanik P. (2011). Synthesis, characterization, and *in vitro* biological evaluation of highly stable diversely functionalized superparamagnetic iron oxide nanoparticles. J. Nanopart. Res..

[34] Vácha R., Martinez-Veracoechea F.J., Frenkel D. (2011). Receptor-mediated endocytosis of nanoparticles of various shapes. Nano Lett..

[35] Atabaev T.Sh., Lee J.H., Han D.-W., Hwang Y.-H., Kim H.-K. (2012). Cytotoxicity and cell imaging potentials of submicron color-tunable yttria particles. J. Biomed. Mater. Res. A.

[36] Chithrani B.D., Chan W.C. (2007). Elucidating the mechanism of cellular uptake and removal of protein-coated gold nanoparticles of different sizes and shapes. Nano Lett..

[37] Jiang W., Kim B.Y., Rutka J.T., Chan W.C. (2008). Nanoparticle-mediated cellular response is size-dependent. Nat. Nanotechnol..

[38] Atabaev T.Sh., Jin O.S., Lee J.H., Han D.-W., Vu H.H.T., Hwang Y.-H., Kim H.-K. (2012). Facile synthesis of bifunctional silica-coated core-shell Y_2_O_3_:Eu^3+^, Co^2+^ composite particles for biomedical applications. RSC Adv..

[39] Sohaebuddin S.K., Thevenot P.T., Baker D., Eaton J.W., Tang L. (2010). Nanomaterial cytotoxicity is composition, size, and cell type dependent. Part Fibre Toxicol..

[40] Park K.S., Lee H.S., Kim Y.S., Kang T.M., Lee J.H., Joh J.W., Kim S.J. (2011). Improved quantification of islet transplants by magnetic resonance imaging with Resovist. Pancreas.

[41] Wang S., Fang J., Zhang T., Wang B., Chen J., Li X., Zhang S., Zhang W. (2011). Magnetic resonance imaging targeting of intracranial glioma xenografts by Resovist-labeled endothelial progenitor cells. J. Neurooncol..

[42] Mahmoudi M., Sant S., Wang B., Laurent S., Sen T. (2011). Superparamagnetic iron oxide nanoparticles (SPIONs): Development, surface modification and applications in chemotherapy. Adv. Drug Deliv. Rev..

[43] Lin M.M., Kim D.K., El Haj A.J., Dobson J. (2008). Development of superparamagnetic iron oxide nanoparticles (SPIONS) for translation to clinical applications. IEEE Trans. Nanobiosci..

[44] Bae J.E., Huh M.I., Ryu B.K., Do J.Y., Jin S.U., Moon M.J., Jung J.C., Chang Y., Kim E., Chi S.G. (2011). The effect of static magnetic fields on the aggregation and cytotoxicity of magnetic nanoparticles. Biomaterials.

[45] Rossi L.M., Quach A.D., Rosenzweig Z. (2004). Glucose oxidase—magnetite nanoparticle bioconjugate for glucose sensing. Anal. Bioanal. Chem..

[46] Blazer-Yost B.L., Banga A., Amos A., Chernoff E., Lai X., Li C., Mitra S., Witzmann F.A. (2011). Effect of carbon nanoparticles on renal epithelial cell structure, barrier function, and protein expression. Nanotoxicology.

[47] Singh N.P., McCoy M.T., Tice R.R., Schneider E.L. (1988). A simple technique for quantitation of low levels of DNA damage in individual cells. Exp. Cell Res..

[48] Cemeli E., Anderson D. (2011). Mechanistic investigation of ROS-induced DNA damage by oestrogenic compounds in lymphocytes and sperm using the comet assay. Int. J. Mol. Sci..

[49] Klarić M.S., Darabos D., Rozgaj R., Kasuba V., Pepeljnjak S. (2010). Beauvericin and ochratoxin A genotoxicity evaluated using the alkaline comet assay: Single and combined genotoxic action. Arch. Toxicol..

